# Resilience amongst Older Colombians Living in Poverty: an Ecological Approach

**DOI:** 10.1007/s10823-016-9303-3

**Published:** 2016-09-01

**Authors:** Kate M. Bennett, Maria F. Reyes-Rodriguez, Paula Altamar, Laura K. Soulsby

**Affiliations:** 1 Institute of Psychology, Health and Society, University of Liverpool, Eleanor Rathbone Building, Bedford Street South, Liverpool, L69 7ZA UK; 2 Universidad El Bosque, Bogotá, Colombia; 3 Facultad Latinoamericana de Ciencias Sociales, México City, Mexico

**Keywords:** Ecological model, Late life, Poverty, Qualitative, Resilience

## Abstract

Older Colombians face significant adversities: poverty, violence and displacement. However, there is evidence that Latinos are often resilient. We examine resilience in older Colombians living in poverty using an ecological framework that identifies three levels: individual; community; and societal. In this paper we examine data from 16 semi-structured interviews with older Colombians that explore resilience within the context of poverty. We analyze our data using three stages: (1) modified grounded theory; (2) assignment of resilience status; (3) identification of components of the ecological framework which contribute to resilience in these participants. The most striking feature is that some participants are able to adapt to their situation, demonstrating resilience, whilst others are not. Individual characteristics such as psychological and material resources contribute to resilience. At the community level, family, social support, participation and cohesion promote resilience. Finally, at the societal level, social and welfare services, finance, religion and social policy, are important factors. These different levels of resilience are co-dependent, and we illustrate how this is so. We suggest that older Colombians living in poverty often demonstrate resilience, but that more can be done to enhance their lives. This includes interventions at the individual and community levels alongside changes in social policy.

## Introduction

Recently, there has been an increasing interest in resilience, and in the ways people ‘bounce back’ from adverse situations (Masten 2007). This contrasts with traditional deficit models that focus on psychopathology and how people are unable to cope with adversity. Much work has focused on children, or adults in Western, industrialised countries (Bennett 2010; Rutter 1999; Spahni et al. 2015). Much less has focused on resilience amongst older adults or in the developing world (Bennett 2015 [for a review]; Donnellan et al. 2014; Eggerman and Panter-Brick 2010; Ong and Bergeman 2004). Researchers have considered extreme or unusual adversity, but few have examined more common adversity (Bonanno 2004; Donnellan et al. 2014; Spahni et al. 2015). We address these deficits focusing on Colombian older adults living in poverty.

Many disciplines use the term resilience but not always in the same way. Masten (2007) suggests three ways that resilience is conceptualised: developing well in high-risk situations; functioning well in adverse situations; and bouncing back after catastrophic situations or deprivation (see also Bennett 2015). Whilst in the past resilience has been seen as uncommon and occurring in relatively few people, more recently studies have demonstrated that resilience is more frequent (Bennett 2010; Bonanno et al. 2004; Donnellan et al. 2014). For example, Bonanno et al. demonstrated that almost half of their sample of widowed older adults were resilient and Bennett found almost 40 % of her sample of widowed men became resilient. In a sample of older British spousal dementia carers Donnellan et al. found that 40 % were resilient. These participants were not wealthy by British standards but were able to access health and welfare services and were all able to access retirement pensions (state-provided and/or employment). In these examples, the challenge faced by adults is not an extreme adversity but more common adversities. Other examples of more common adversity include family breakdown, the challenges of later life, ill-health, and poverty (Becker and Newsom 2005; Eggerman & Panter-Brick; Ungar 2010; Windle and Bennett 2011). Thus, exploring resilience among people facing late life poverty, common in both in the developed and developing worlds, is an important area of study.

As we have noted, resilience has been conceptualised in different ways (Masten 2007). To address some of these issues the Resilience Network (of which Bennett is a member) examined how resilience can be developed, maintained and enhanced to reduce health and social inequalities and achieve healthy ageing across the life-course (http://resilience.bangor.ac.uk/). As part of this work, Windle (2011) conducted a conceptual review of resilience across the lifecourse. She proposed the following definition (p. 163):

Resilience is the process of effectively negotiating, adapting to, or managing significant sources of stress or trauma. Assets and resources within the individual, their life and environment facilitate this capacity for adaptation and ‘bouncing back’ in the face of adversity. Across the life course, the experience of resilience will vary.

In this paper we adopt this definition because it highlights both the lifespan and external factors as being important in understanding resilience.

**Fig. 1:**
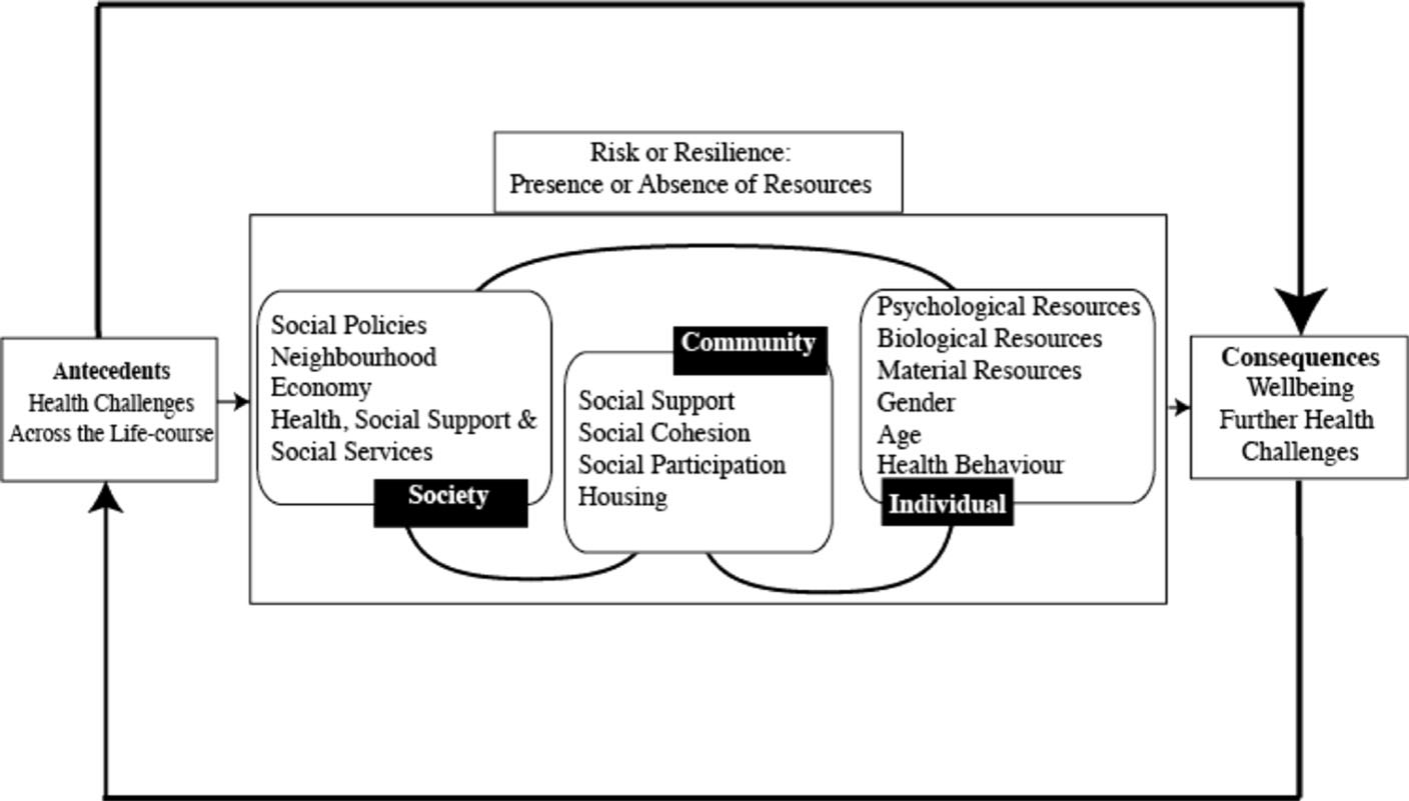
Ecological model of resilience. Figure shows how resilience develops from the antecedents, such as poverty, with foci on the individual, community and societal levels. (adapted from Windle and Bennett 2011)

Resilience has been examined on different levels (individual, community, society); most frequently at the individual level (Ong et al. 2006), but this neglects the interplay between different levels. However, an ecological systems approach identifies and examines inter-level interactions (Bronfenbrenner 1994). One of the strengths of Windle’s (2011) definition of resilience is that it puts resilience within such an ecological framework, emphasising how resilience operates, interactively, at the level of individuals, community and society (Ungar 2011). Whilst this framework is often used in child development, it is becoming more familiar in gerontology (Bennett 2015; Donnellan et al. 2014; Ong et al. 2009). As Ungar (2011) points out, context is crucial: individuals may fail to become resilient if the community does not facilitate opportunities to adapt. Wiles et al. (2012) also highlighted the importance of environmental-community and social-political structures in fostering resilience in later life. As part of the Resilience Network, Windle (2012) developed an ecological framework of resilience across the lifespan (see Fig. 1) and in the context of familial caregiving (Donnellan et al. 2014; Windle and Bennett 2011). Although the authors of these papers are British the ecological model was developed from an extensive conceptual analysis and literature review which was international in scope and not confined to Britain. One of the aims of this paper is to examine whether the framework is applicable to resilience in the context of poverty.

One of the key features of the ecological framework of resilience is its emphasis on the factors and resources that contribute to resilience. At each level, individual, community and society, it identifies the resources individuals may have access to and on which they might draw. At the individual level, psychological resources, such as mastery and perceived control; biological resources, including good health, genes and health behaviours such as exercise and diet; material resources, including access to housing, food and money; and age and gender influence resilience. At the community level factors such as social support, participation and social cohesion might be important as well as the housing environment. Finally, societal resources could include social policy, social, health and welfare services. As we have already mentioned the levels interact with each other to contribute to resilience or further risk.

One of the challenges people face, especially in the developing world, is poverty. Poverty threatens autonomy and personal development. It can prevent the exercise of social and civil rights and disrupt social belonging (Corredor 2004; Garay 2002). It is also a risk factor for ill health and impacts negatively on both the individual and wider society (DESA-United Nations 2010; Oxford Poverty and Human Development Initiative, OPHI 2010). However, there is evidence that some people living in poverty are resilient. Eggerman and Panter-Brick (2010) found that one of the main stressors identified by Afghani children and their caregivers was economic uncertainty. This triggered insecurity and the perception of loss of honour. They found that both family life and strong religious values promoted resilience in these circumstances. These studies suggest that poverty, whilst it may be commonplace, nonetheless is an important stressor, which can lead either to a failure to cope or to resilience. Further, the influence of poverty may differ across the lifespan both in terms of the experience of poverty and in terms of availability of finance. It is likely that older adults experience poverty differently to children or people at other stages of the lifecourse and older adults may become poor, or poorer, when they cease work or when they seek work, or when a spouse dies, for example (Lloyd-Sherlock 2000). Old age poverty has been identified as a problem and, in Latin America, the incidence of poverty is generally higher amongst older adults compared to the population as a whole (Barrientos 2008).

Levels of poverty in many parts of South America are higher than in the developed West (World Bank Group 2015). Yet, Latinos are often believed to be resilient regardless of where they live (Ong and Bergeman 2004). Factors which promote resilience amongst this population include the cultural and moral values associated with family, strong social networks and religious beliefs (Gallo et al. 2009; Markides and Eschbach 2005).

Cárdenas and López (2010) developed a theoretical model of resilience developed in Colombia (the Analysis Matrix of Resilience) which identified 6 inter-related factors promoting resilience: political, social and institutional organization; cultural beliefs; environment; coping; individual characteristics; and social networks. These components resemble the ecological framework shown in Fig. 1. Cárdenas (2008) found that the experiences of older Colombians mapped onto this matrix.

Colombia has a population of more than 43 million (Departamento Adminstrativo Nacional de Estadístsica – DANE [National Bureau of Statistics] 2005). According to the World Bank, 29 % of the population were living below the UN’s poverty line (and at the time of the study this figure was 46 %) (US$2 per day or US$60 a month: PovcalNet 2014), and this figure is also representative of Bogotá’s population. At the time of the study 18 % were living in extreme poverty, although this figure is now 8 % (Departamento Administrativo Nacional de Estadística – DANE 2015). In Bogotá, 10 % of the population were 60 years old and over (Altamar 2006; SDIS 2008), and about 40 % are living in poverty.

In this paper we examine resilience in older Colombians living in poverty using data collected as part of wider study of wellbeing and poverty. Participants were not asked directly about resilience. Thus, a key feature of this paper is that the data on resilience emerged spontaneously and Becker (1958) argues that participants’ volunteered statements reflect their concerns more accurately than interviewer directed questions (Bennett and Soulsby 2012). The sample is larger than has previously been used in qualitative studies of poverty in Colombia (Cárdenas 2008). Qualitative data allows us to capture the subjective daily experiences of older people living in poverty which may be missed by questionnaire data. Camfield et al. (2009) recommend the use of qualitative methods when examining wellbeing and quality of life of people in developing countries because they take account of the context and the ways in which people conceptualise their wellbeing. We address four questions. First, is this sample of older Colombians, living in extreme poverty, resilient? Second, is the ecological approach an effective framework to aid our understanding of resilience in the context of poverty in this sample? Third, if the framework aids our understanding of resilience, how do the resilience factors and resources identified in the context of poverty map onto the factors in the framework outlined in Fig. 1? Finally, how may resilience be promoted in older people who are not yet resilient?

## Design and Methods

This paper draws on the analysis of qualitative interviews with 16 older Colombian adults, collected by Reyes-Rodriguez and Altamar in 2009 as part of a wider study. The wider study explored perceptions of wellbeing, independence, and health, within the context of poverty. The data from the wider study was analysed using modified grounded theory (Bennett and Vidal-Hall 2000). See below for more details on how the analysis was undertaken). A central theme within the data was resilience and that provides the focus for this paper.

### The Researchers

The original work was part of Altamar’s PhD project, where she was developing an index to assess public policies in Colombia and Reyes-Rodriguez was her Research Assistant conducting the interviews. Reyes-Rodriguez is a Colombian Psychologist who has experience working with the older population living in poverty in Bogotá, and has conducted qualitative interviews for various projects; she has over 4 years experience. With permission to use the data from Altamar, Reyes-Rodriguez undertook an MSc under the supervision of Bennett focusing on resilience that forms the basis of this paper. Bennett, Reyes-Rodriguez and Soulsby are Psychologists who focus on ageing, wellbeing and resilience. Bennett and Soulsby have expertise in qualitative methods (20 years and 10 years respectively and with more than 25 qualitative papers collectively). Altamar is an Economist with an MSc in Social Policy.

### Participants

The participants were recruited from a project of the city government of Bogotá, Atención Integral para la Garantía de los Derechos para una Vejez Digna en el Distrito Capital [Integrated Care for the Assurance of the Right to Age with Dignity in the Capital], that aimed to improve quality of life by providing social services to older people who were not receiving a pension and were living in extreme poverty and homelessness (Secretaría Distrital de Integración Social, SDIS [Secreatriat of Social Integration] 2008). Hereafter it will be referred to as the Project. It provides a monthly allowance and facilitates social participation by training community leaders, creating leisure groups, and holding workshops for older people. It offers care home places to older people in extreme poverty (Altamar 2006; Departamento Administrativo de Bienestar Social -DABS 2006; Secretaría Distrital de Integración Social, SDIS [Secreatriat of Social Integration] 2008). However, the Project only covers 17 % of the eligible population (Secretaría Distrital de Integración Social, SDIS [Secreatriat of Social Integration] 2008).

Recruitment to the project was as follows: any older person living in Bogotá could express interest in the project, or alternatively they could be referred by community organisations; next a team member from the Project verified whether the person met the eligibility criteria which were the following:

1. Not receiving a pension;
2. To be classified by the SISBEN (Sistema de Identificación y Clasificación de Potenciales Beneficiarios de los programas sociales - SISBEN [Identification and selection system of social programs beneficiaries]) as meeting its lowest levels (1 or 2), representing vulnerability and poverty (Departamento Nacional de Planeación – DNP [National Planning Bureau] 2008);
3. To have an income less than the minimum wage of $300 USD per month;
4. Live in a family with an income below the minimum wage;
5. To be homeless and living on public charity.

Only those who met at least the first two criteria were eligible to receive the monthly allowance or to be on the waiting list. Active participation in the Project was dependent on the availability of places. In Bogotá approximately 270,000 older adults are classified as levels 1 or 2 of SISBEN (Secretaría Distrital de Integración Social, SDIS [Secreatriat of Social Integration] 2008). Nearly 35,000 older people are beneficiaries of the Project, 10,000 are on waiting list and 30,000 have expressed interest but are not yet on the waiting list. The monthly allowance does not guarantee that older adults are no longer in poverty or homeless. The Project does provide social activities and workshops which can be accessed by people not meeting the criteria, especially if they receive a pension from elsewhere.

In the original study, Altamar developed a sampling frame (2006) using a principal component analysis, to establish poverty profiles, based on the work of Sen (1995). Sen argued that it was important to take account of a person’s *functioning* and *capabilities* when studying the relationship between poverty and wellbeing (Nussbaum and Sen 1993). *Functioning* refers to the active realisation of a person’s achievements and goals and *capabilities* to the abilities and options the person has that facilitates the achievement of those goals (Drèze and Sen 1991; Jean and Sen 1991; Nussbaum and Sen 1993; Nussbaum 2011; Sen 1995). Thus, we need to take account of the variations in poverty. A categorical principal components analysis (CAPTCA) was undertaken using the Project database followed by optimal scaling analysis (Altamar 2006). 56,440 people were registered on the database, of whom 38,406 were receiving the monthly allowance and 18,034 were on the waiting list (Altamar 2006). The CAPTCA was performed on the following categorical variables recorded by The Project: sex, age, marital status, economic status, disability, whether engaged in formal or informal work, relationship to head of household, whether they have children and whether that relationship is active, SISBEN score and current activity. Seven distinct dimensions emerged from the analysis, explaining 84 % of the variability (Altamar 2006; Reyes et al. 2014). A brief description of the dimensions, and where participants matched onto them, can be found in Table 1.

**Table 1:** Sampling frame dimensions obtained by CAPTCA and optimal scaling and participants that represented each dimension

Dimension	Dimension description	Participants
1	Explained 14.3 % of variability. Two profiles were identified, i. characterised by marital status, living with someone, male, age 60–69 years. ii. widow, women, SISBEN 3.	Mr. 5 Mrs. 7 Mr. 14
2	Explained 13.6 % of variability Two profiles were identified, i. people that do not work, women, do not have an active relationship with children. ii. work, active relationship with children, men.	Mrs. 15 Mr. 3 Mr. 4
3	Explained 10.6 % of variability. Two profiles were identified i. head of household, men, highly vulnerable (level of SISBEN 0 or 1). ii. SISBEN 2, partnered.	Mr. 3 Mr. 8
4	Explained 9.4 % of variability. Disability and suspected to be a victim of domestic violence	Mrs. 1 Mrs. 2
5	Explained 9.3 % of variability. Receives monthly allowance and highly vulnerable (level of SISBEN 0 or 1)	Mr. 8 Mrs. 9.
6	Explained 9 % of variability. Receives economic support (different from the allowance), pensioned, waiting list	Mrs. 6 Mrs. 13
7	Explained 8.9 of variability. Receives economic support (different from the allowance)	Mr. 10 Mr. 11 Mrs. 16

Altamar (2006) stopped the analysis in dimension eight because the variables that explained the variance were the same as dimension four. The table was adapted from Altamar (2006) and Reyes et al. (2014)

The Project Directors were asked to identify participants who represented each of the dimensions in the sampling frame (see Table 1). To recruit a minimum of 12 participants, 16 adults were invited to participate. However, all 16 agreed to take part and were, therefore, interviewed. 8 women and 8 men aged 60 years + (mean = 69.8 years, range 60–86) were interviewed. Guest et al. (2006) suggest that 16 interviews should be sufficient for theme saturation. Participants were either participating in the Project or on the waiting list. 12 were participating in the Project and received a monthly allowance (USD$40) (less than USD$2 per day). Three participants were on the waiting list and not receiving the allowance. One woman was a pensioner, who received the legal minimum monthly salary (USD$271) as part of her pension, was not receiving the Project allowance but was on the waiting list.

### Interview

Semi-structured interviews were conducted to identify the subjective wellbeing of the participants. The following aspects were explicitly explored: (a) perceptions of wellbeing; (b) independence; (c) social support; (d) perception of health; (e) religion; (f) work, social activities and leisure; and (g) income and finances. Questions included: “How do you feel about your life right now? Why?” “What you like about your life? Why?” “What do you dislike?” “Tell me more about the activities you are involved in? What does the allowance mean to you? ” “Why do you feel at peace with yourself?” “You tell me that you are thankful to God for what has happened to you, please tell me more about it. How does this belief in God help you?” (full interview schedule available on request).

All interviews were conducted in Spanish by the second author Reyes-Rodriguez and lasted thirty-sixty minutes. All the interviews were voice recorded and transcribed into Spanish, and coded by Reyes-Rodriguez and Altamar. A quarter of the interviews were translated into English and coded by Bennett. All quotes were translated into English. Note that some of the quotes appear to be worded awkwardly to the English ear. This arises from two issues: first, participants had low levels of education and, therefore, in the Spanish transcriptions, the participants did not speak in grammatically correct ways and used idioms; second, to preserve the sense and feeling of the interviews, we have chosen to present literal rather than grammatically correct quotes.

### Ethical Considerations

An agreement was established with the Secretariat for Social Integration of the city government of Bogotá. Staff on the Project facilitated recruitment in 5 of the twenty boroughs of Bogotá. The participants were contacted directly by telephone or at the boroughs’ community facilities. The purpose of the interview was explained to participants and anonymity and confidentiality assured. Participants were assured that participation was voluntary, that they could withdraw at any time, and that their position in the project would not be at risk, regardless of whether they participated or not. Participants gave audio-recorded verbal consent. The interview was conducted at the closest local borough facility to the participant. Permission to use the data was given by the District Secretariat for Social Integration of the city government of Bogotá. Subsequently, The University of Liverpool Research Governance Committee was informed about: (a) the origin of the data; (b) the official permission given by the District Secretariat for Social Integration of the city government of Bogotá; and (c) the confidentiality of the data. The University of Liverpool Research Governance Committee approved the ethical arrangements.

### Data Analysis

We adopted a three-stage hybrid method of analysis, which has been employed elsewhere (Donnellan et al. 2014).

Stage 1: The interviews were analysed using Bennett and Vidal-Hall’s modified grounded theory (2000). Firstly, interpretive memos were created describing first impressions and the general characteristics of the interview. Secondly the transcripts were read and re–read line–by-line and coded. The initial coding was attached closely to the data and the codes were open-ended to facilitate the emergence of new ideas (Charmaz 1995; Bennett and Vidal-Hall 2000). The most common themes were identified and 164 codes emerged. The most common theme was that of resilience, and this forms the focus of this paper.
Stage 2: We identified participants as resilient or not resilient. We did this through careful reading of the interviews by 2 members of the team (Reyes-Rodriguez and either Bennett (25 % of the interviews) or Altamar (75 %). In addition, Bennett examined all quotes and categorised these quotes as demonstrating resilience or not. We used Windle’s definition of resilience (see above), which was operationalized, thus:
  i. There must be a significant challenge, and here this was poverty
  ii. No sign of (dis)stress or negative outcome
  iii. Maintaining a life of meaning and satisfaction (a sign of bouncing back)
  iv. Actively participating in life (a sign of managing)
  v. Current life seen as positive (a sign of adaptation)

Using this operationalisation we identified 4 participants as not resilient and 12 as resilient. This follows the same strategy as Donnellan et al. (2014). Consensus was arrived at through discussion (see also Trustworthiness below). See below for two detailed examples of classification.

Stage 3: We reanalysed the interviews focusing on resilience, refining our coding. From this more focused analysis fourteen themes emerged which included psychological, material, social support, religion and the interplay between age and gender. We examined to what extent these themes mapped onto the ecological framework of individual, community and societal factors. We also focused on how one could promote resilience using these factors.

#### Trustworthiness

There are challenges in conducting cross-cultural work where interviews are conducted in one language (Spanish) but presented in another (English), and where the authors come from two different cultures (Reyes-Rodriguez and Altamar: Colombian; Bennett and Soulsby: British). However, this is also a strength since it requires all authors to understand each others’ perspectives. Cultural-specific concepts need to be explained clearly to those unfamiliar with culture. Thus, developing themes were agreed through a process of discussion and consensus.

## Results

In this paper we address four questions. First, are older adults among this sample of participants, who live in poverty, resilient? Broadly speaking the answer is yes. We classified 75 % of our sample as resilient. Second, is the ecological approach an effective framework for understanding resilience in the context of poverty in this sample? Third, if the framework aids out understanding of resilience, how do the resilience factors identified in the context of poverty match onto the factors in the framework outlined in Fig. 1? We find that our data map onto ecological framework in terms of individual, community and societal factors. However, we find that there are some additional factors to be added to the ecological model, and conversely some factors present in the original model for which are not supported by the findings of this study. For ease of reading we address questions 2 and 3 together. Finally, we ask how may resilience be promoted in older people who are not yet resilient? We address this issue in our discussion.

### Are Older Adults among Sample Participants Who Live in Poverty, Resilient?

Table 2 shows the demographic characteristics of the participants. Of the 16 participants, 12 were identified as resilient and four were not. In the non-resilient group, the two women participants were active in the Project and the two men were on the Project’s waiting list and were not receiving the monthly allowance. Three participants had been exposed to additional stressors. One man was forcibly displaced (Mr. 3) and the two women were suffering either from disability or illness (Mrs. 1 and Mrs. 2). The fourth non-resilient participant was Mr. 4. Within the resilient group there were also participants who had been exposed to additional stressors: Mr. 14 was awaiting surgery and Mrs. 13 was caring for a sick daughter and was not receiving the allowance. Thus, it was not an accumulation of stressors that contributed per se to a lack of resilience.

**Table 2:** Socio-demographics characteristics of the participants

Participant Age		Marital status	Illness	Occupation	Project	Resilient
Mrs. 1	84	Widow	Yes	None	Active^a^	No
Mrs. 2	70	Single	Yes	None	Active^a^	No
Mr. 3	71	Divorced	No	Casual work	Waiting list^b^	No
Mr. 4	64	Single	No	Casual work	Waiting list^b^	No
Mr. 5	68	Married	No	Casual work	Active^a^	Yes
Mrs. 6	70	Widow	No	Leisure^d^ /pension	Clubs^c^	Yes
Mrs. 7	69	Widow	No	Community leader	Active^a^	Yes
Mr. 8	72	Widower	No	Leisure^d^	Active^a^	Yes
Mrs. 9	65	Married	No	Leisure^d^	Active^a^	Yes
Mr. 10	86	Married	No	None	Active^a^	Yes
Mr. 11	73	Married	Yes	None	Active^a^	Yes
Mr. 12	66	Married	No	Leisure^d^ /Casual work	Active^a^	Yes
Mrs. 13	60	Married	No	Casual work	Waiting list^b^	Yes
Mr. 14	69	Married	No	Community leader	Active^a^	Yes
Mrs. 15	69	Single	No	Leisure^d^	Active^a^	Yes
Mrs. 16	60	Single	Yes	Casual work	Active^a^	Yes

^a^Active: receiving the monthly allowance

^b^Waiting list: Not receiving the allowance

^c^Participates in leisure activities and is not receiving the allowance

^d^Participates in leisure activities

We give two detailed illustrations of how we classified our participants. Mr. 8 was classified as resilient because he showed no obvious sign of distress. He had adapted to a life of poverty, and viewed his life positively:

Currently I feel fine, I do not feel bored, I do not feel afflicted, because you have to try to get ahead, because some people may feel bad. You need to get ahead and be active.

On the other hand Mrs. 1 was not resilient because she is distressed, not positive about her circumstances and is unable to participate fully in life.

Well, because of the years I have and who knows until when God will remember me, and I feel sad and feel alone. And well I don’t know if my children will be able to bear with me and help me anymore, and I hope that welfare (The Project) will not leave me.

### Is the Ecological Approach an Effective Framework for this Sample and how Do Resilience Factors Map onto the Framework Outlined in Fig. 1?

Overall we find that the factors that emerge from our analysis map well onto the ecological framework at individual, community and societal levels. We discuss factors at each level in turn. We highlight where new factors are relevant and also identify factors in the framework which did not emerge in our analysis.

### Individual Level of the Ecological Model of Resilience

Four individual factors were noticeable in distinguishing the resilient participants from those who were not: psychological resources/factors; material resources; biological resources; and gender and age.

### Psychological

We found several psychological factors common to our resilient participants. The first was mastery. Mr. 14 suggested that his ability to deal with what life threw at him enabled him to thrive, alongside an awareness that there would always be problems that would need to be confronted:

My life hasn’t been easy, but I know how to deal with that… problems will always exist…it is not that because you don’t have money, you’ll feel ashamed, no, no. (Mr. 14 Resilient)

Other participants demonstrated life-long optimism, as illustrated by this man:

You have to be active! Even if you are sick, say no! I’m blessed! Even if a knee is hurting, say to yourself “I am healthy”. Not like other people, that when are asked "what do you feel?" they answered "Sick .... Sick ...” , well then called over the disease, one has to be active, and draw forces from you don’t have. (Mr. 10, Resilient)

A sense of control was apparent amongst the resilient participants. Mrs.7 suggested that having control over what she could cook was important:

Very important (the allowance), because I don’t have to depend on what my daughter is going to cook for lunch, because I know what I have and what I can do today, what I have to cook for tomorrow.

In contrast, a lack of control contributed to Mr. 3’s lack of resilience. He was forcibly displaced by guerillas and talked about how his life had changed from one in which he was in control to one in which he no longer was, and this made him unhappy:

A horrible thing, the sadness is killing me, because I used to live where I lead, where I decided everything… because what had happened is that I have to wait till 1 or 2 p.m. to drink a cup of coffee, or chocolate, I have to wait for people’s good will. (Mr. 3, Non-resilient)

Control is important for resilience in two ways. First, for most participants there is an objective lack of control through their lack of money. At the same time, what appears to be more important in terms of whether participants are resilient or not is perceived control. Mrs. 7 has only the allowance, less than 2 US$ a day which does not give her much objective control over her life, but she perceives and exercises control with her limited budget in determining what she can eat.

### Material Resources

The second individual factor influencing resilience is the presence of material resources. Participants spoke most often of how money allowed them, or prevented them, from paying the rent. In the case of Mr. 8, his resilience was enhanced because he had money that allowed him to buy things for the house:

Because I receive money as well, I say ‘Ok, what we are missing in the house?’ What do we have to buy? (Mr. 8, Resilient)

He demonstrated how being able to buy things allowed him to exert control over his life. Two resilient women talked about the importance of money to pay the rent. In both of these cases, the money came not from the Project but from other sources. Mrs. 13 said:

…then he (ex-boss) gave us a small house where to live, therefore, in that house there is like a small flat on the ground floor that is being rent out. We use that money to pay the food and bills. (Resilient)

In contrast, two of the non-resilient participants spoke of how the lack of money to pay the rent made them either ill or unhappy. Mr. 4 said:

…I feel desperate, yes. That is making me sick, not having money; for example, soon I have to pay the rent and I don’t have money, then ... I don’t sleep, because I keep thinking. (Non-resilient)

Mr. 8, although resilient, identified the problems of being without money for:

Being without money is sorrow, you feel bad when you don’t have any money in your pocket, to say I’m going to buy something…(Resilient)

Whilst these material resource issues function at the individual level, they also function at the community level such as housing (in)security. Mrs. 13 was provided with housing security and social support from her ex-employer. Further, since there were relatively few social policies that protected older people from financial insecurity or homelessness, it was also possible to see this as a societal level issue. It is also important to bear in mind that the participants were living in poverty. All but one either received the allowance or were on the waiting list, and thus received no money. All but one of the participants received less than the poverty line of the UN’s 2 US$ a day.

### Biological Resources

Resilience was enhanced by biological resources, which primarily concerned physical health. Many participants argued that good health was the key to wellbeing. Mrs. 16 summed up this view:

Health is to be able to walk, to see, to talk; to be able to eat, to do exercise, to be able to do everything… without asking favors to anybody, not even my family. (Resilient)

Resilience could be, at least temporarily, threatened by short-term anxieties about health, as outlined by Mr. 14:

At this moment I’m stressed because I will have a surgery. Then I think if I have to be one or two months recovering, I will feel uncomfortable because I will not be able to be with the group. (Resilient)

For some participants permanent health issues contribute to a lack of resilience:

The only thing that bothers me is that I cannot go out by myself like I used to do. (Mrs. 2, Non-resilient)

Mrs. 2’s poor health reduced her sense of personal control and, combined with the lack of social support, inadequate neighbourhood infrastructure and a lack of welfare services, contributed to her lack of resilience.

### Gender and Age

In the original framework age and gender were identified as separate factors. However, in our data these two factors were interlinked. When participants were asked about what growing older meant to them there were gender differences amongst those who were resilient. Whilst the women associated ageing with new freedoms, men found it difficult to accept their age. Mr. 14 associated wellbeing with feeling young:

We have to pretend like if we were young, like fifteen years, twenty (Resilient).

Moreover, the male participants suggested that success was associated with money and recognition, but they found those difficult to achieve in later life. Mr. 5 said:

Now with the years I have, you don’t advance at all, so you have to be like this, because there is nothing else. I mean I can’t progress, I don’t have enough to start up a business and progress more. (Resilient)

On the other hand, the resilient women perceived aging in three ways: as the best time of their lives; (b) associated with freedom; and (c) as a time where they could think about themselves. When we asked Mrs. 15 “what do you like about ageing?” she replied:

Freedom! It’s the most beautiful thing I have. (Resilient)

She found that after a lifetime working as a live-in maid, it was only when she stopped working that she could begin to care about herself. Mrs. 7 said that after becoming a widow and attending the Project, her life changed:

I feel more secure in everything I do, in what I see, in what I feel… I started to feel like a bird that started to fly; because before I was next to my husband, a man who loved me, and I loved him for 40 years, 4 children; but it was 40 years of standing all his drunkenness. (Resilient)

Thus, for women, old age was liberating, whilst for men, old age was a potential threat to their resilience. This threat seemed grounded in men’s perceptions of losing their place in the world.

We note that although health behaviours appear in the original framework it does not emerge in our data. There are two reasons why this may be the case. First, questions about health focused on perceptions of health rather than health behaviours. Second, it may be that in order to enact health behaviours, such as exercise or diet, one needs to have a standard of living that permits the opportunity to think about these things.

### Community Level

Four factors contributed to resilience at the community level: family; social support; social participation; and social cohesion. We deal with family first in part because of the significance of family to Latinos (Cárdenas and López 2010) but also because it was the most important in our analysis. It is interesting to note that family was not specifically identified in the original resilience framework, although it has also recently also been highlighted by Donnellan et al. (2014).

#### Family

The data demonstrates how non-supportive family contribute to a lack of resilience. Mr. 3 represents the clearest example of this, and these two quotes were especially relevant:

Well I’m with my son but I’m not ok there. After you become old you are a nuisance everywhere.

It is pretty bad, because I’m unprotected and all alone. Dr., my daughters, one is married and is in Tolima, but I don’t get along with my sons-in-law, …how I will be there? How I’ll go to live where the son-in-law don’t like me? … the help of nobody, … living with my son where I’m not welcome. (Non-resilient)

Mrs. 1 was suing her family and said:

I’m locked up here and alone. …apparently they are going to take me to other place, I don’t know, who will receive me... is that none of them, that’s why I sued them… (Non-resilient)

Conversely, resilient participants stressed the importance of their families, not only in terms of the support they received from them, but also in the support they give to their families:

I am the one that is looking after them (grandchildren), because their mother works. (Mrs. 9, Resilient)

For me (family) is very important, because after I became a widow 9 years ago, then I had the support of my children were found. (Mrs. 7, Resilient)

But family meant more than support. Family provided identity and status. The resilient men saw themselves as the head of the family, both financially and in status, as Mr. 14 said:

At least you get 80.000 pesos (the allowance – USD$40), and well at least you can give money to buy groceries, paid utilities… it is important to not waste them because that is for food and rent. (Resilient)

Mr. 14 continued:

My son, he knows I like that (community leadership), so he feels proud of me….

Mr. 8 echoed the importance of being head of the family:

I am the head, while you’ll exist, you are the head of the family. (Resilient)

For others, such as Mr. 5, it was the emotional and long-standing bond of marriage that contributed to resilience:

At this age, we have just each other. (Resilient)

The presence of family, the positive support and the role it provided contributed to resilience. On the other hand, lack of family and familial support or poor family relations was detrimental. Thus, it was not only the presence of family but also the quality of familial relationships that were important in determining resilience.

### Social Support

As we have shown in relationship to family, social support was an important contributory factor in promoting resilience. However, social support was drawn from broader sources, both with respect to friendships but also with lasting ties, such as Mrs. 13’s support from her past employer, reported above. Mr. 14 highlighted the importance of support from friends, the need for conversation and the reaffirmation of identity:

Having the friends of my group is… ah, I feel young in the group… I feel like a teenager, I talked with them, I dialogue, and then all of that fills me with satisfaction. (Resilient)

On the other hand, those without social support were less likely to be resilient, as illustrated by Mrs. 1:

I’m suffering a lot, but I ask God to not let me live many years, because there is nobody that can look after me (Non-resilient)

### Social Participation

The same social activity or relationship often serves more than one purpose and this is true in the context of resilience. We have already shown that some familial relationships can be supportive and the same is true for friendships. When we look at social participation, we find further linkages. As the majority of our participants were engaged in the Project, they were engaged in social participation. Mr. 10 illustrates the relationship between social participation and social support:

We meet there all the best friends … when we get hungry, we said ‘today is your turn the mid-morning snack’, so we go to the shop and buy a soft drink, bread, and tomorrow is other one’s turn, and the day after tomorrow to other. (Resilient)

Mr. 8 talked about the way that participation motivated him:

We have a group of ‘tejo’ (traditional Colombian game)…. So that motivates me a lot. Because I direct the group and I feel happy because I am doing a good labour… (Resilient)

In contrast, although Non-resilient participants described themselves as active persons they reported that they were not engaged in activities. As Mr. 3 suggests, the lack of participation affected their physical and mental health:

Oh my God I’m getting ill of not doing anything. (Non-resilient)

Another Non-resilient participant agreed:

When you are not doing anything, a week passed and nothing happened... there is nothing to wait for (Mr. 4, Non-resilient)

### Sense of Community

Participation in the Project also provided a sense of community, especially where participants had become community leaders. It was most clearly illustrated by Mrs. 7:

It is very important for my life. Because through them you learn a lot of things, through people I know, if I know more important people, I can help more people … I come here frequently, to obtain information… then I have a commitment that I need to continue doing it. (Resilient)

Mr. 14 also worked as a volunteer and commented:

I feel proud of myself and satisfied for that. (Resilient)

The sense of community was not present in the original resilience framework although social cohesion was.

It was clear that the resilient participants were socially active, and this participation enhanced their motivation to develop social networks. Further, it gave participants a purpose in life and protected them from boredom and sadness. Those who were not resilient, in contrast, found themselves isolated and this impacted on their physical and mental health. Participation in the Project contributed to resilience and this is a potentially confounding factor. However, it was not the case that participation in the project was a necessary condition for resilience. Half of the non-resilient participants were engaged with the Project, and two of the resilient participants were either on the waiting list or not in receipt of the allowance. In future work it might be interesting to look at a sample of participants on the waiting list and compare them more systematically with those already engaged on the project to examine the role of the Project itself in promoting resilience.

### Societal Level

The most obvious example of how resilience could be fostered at the societal level was the Project, an important aspect of the social and welfare services provided by the city of Bogotá. There were other societal influences that had the potential to promote resilience such as social policy. In addition, the religious underpinnings of Colombian society were also important. On the other hand, other societal factors had the potential to undermine resilience, such as violence and displacement and poverty itself.

### Social and Welfare Services

We examine the role of the Project itself. For Mrs. 15, the Project director had become family:

I have found like a mom in Mrs. Y. (Resilient)

On a more practical level, Mrs. 7 and Mr. 10 identified the financial support that the Project provided as a key feature contributing to their resilience:

…very important (the allowance), because is my Money, with that Money I don’t have to ask my daughter I say ‘look I have this money’, No!, with this money (the allowance) I received, I buy what I want. First I have to buy something to eat, and I can say that I’m going to do some “arepas” (like corn cheese pasty), and if can I’ll go and buy a scarf. Yes it is very important for me this money (the allowance) (Mrs. 7, Resilient).

It is a help [the allowance] for self-sustaining but not in large quantities, but it is important to know how to enjoy every penny, if you do it could be enough. The money is not for spending in just one day. (Mr. 10, Resilient)

Although earlier we discussed finance in terms of a material resource, it is clear that it is also an important societal resource, in this case, directly provided by the Project, and thus functions at two levels, which was not considered in the original ecological framework.

### Religion

A strong theme in the data was the role played by religion and faith in resilience, and again this did not appear in the original resilience framework. The belief in God provides a life view framework that is strong in Colombian society (Reyes-Ortiz et al. 2007). The resilient participants were grateful to God. For example Mrs. 7 said:

We have to be grateful with God, with what he is giving us and with what the government if giving us. *(*Resilient)

Participants believed that their faith was rewarded with material assistance and this appeared to promote resilience. Mrs. 13 believed that her house was a gift from God:

Well, she (Virgin Mary) helps a lot, she, the Virgin did the miracle that we have been given the house, it was to her I prayed and asked for it. (Resilient)

Others in the resilient group avoided asking God for more even though they were still aware that they needed more. There was a sense in which their destiny was in the hands of God:

Well, no, I don’t ask anything else to God, what I already have is very big (Mrs. 13, Resilient)

For those who were not resilient, religious beliefs were often the only source of hope; despite their distress and suffering, they referred to belief in God’s will and mercy. Mrs. 2 articulates this:

God takes care of you; God has mercy;

Mrs. 2 continued saying:

I believe in God and hold the hands of God; because now I … you lost friendships, now you not even have any friends… the friends are just few. (Non-resilient)

Religious beliefs helped the resilient group to cope with their poverty. Their sense of destiny, and their belief that their situations were “God’s will” were important. This was true not only for the Resilient but also for the Non-Resilient.

### Social Policy

The Project is an example of how social policy can promote resilience. However, there is one example of where the interaction between social policy and the individual was unsuccessful. Mrs. 2 was one of the Non-resilient participants, she was living in extreme poverty and was considered to be at a high risk and vulnerable. However, she was not willing to leave her house, or allow somebody to help improve her living conditions, even though this was a realistic option.

Mrs. 2. I thought one day, God, ergh! But I cannot resist this, and evil thoughts came, of killing myself, to take my own life… see I have the water cut, gas cut... more or less 3 to 4 months ago. (Non-resilient)

This resonates with findings from Donnellan et al. (2014) who found that not only was the availability of resources important in facilitating resilience but also important was the willingness to access those resources.

### Violence and Displacement

Alongside poverty one aspect of Colombian society that distinguishes it from North America or Europe is the frequency with which people face violence and displacement. As the original framework (Fig. 1) was developed in the UK, violence and displacement was not included in the original formulation. Mr. 3 describes how these experiences have influenced his life, and have contributed to his lack of resilience. He had experienced a productive, rural life until he got on the wrong side of the guerillas:

I achieved some things there (countryside), and I had a good time, and then I was kicked out by the guerrillas, and then I had nothing to eat (Mr. 3, Non-resilient)

Collectively, societal level factors contribute to the development of resilience or hinder its development. Social policies, cultural and historical influences such as religion and violence, are all influential. However, their influences on resilience are best understood in their interplay with individual and community levels. Note neither neighbourhood nor the economy emerged as factors spoken about by participants in our analysis. However, the economy is important, at the macro level, since it is fundamental to issues of poverty.

### Cumulative Stress

Outside of our discussions of the ecological framework of resilience, another issue warrants some comment and analysis. In the methods we note that poverty and increasing age were not the only stressors faced by our participants. A number of our participants faced additional health, family or displacement issues. This was true of half of our small resilient sample, and the proportions were less amongst the resilient sample. Researchers are beginning to examine the influence of cumulative adversity on resilience (e.g. Seery et al. 2010). Seery et al. (2010) suggest a u-shaped curve where those with some lifetime adversity had higher wellbeing than those with high levels of life-time adversity or none. In the current study it is difficult to determine the precise impact of such stressors, since we are reliant on participants mentioning them spontaneously. However, it would be an important area of future research.

## Discussion

**Fig. 2:**
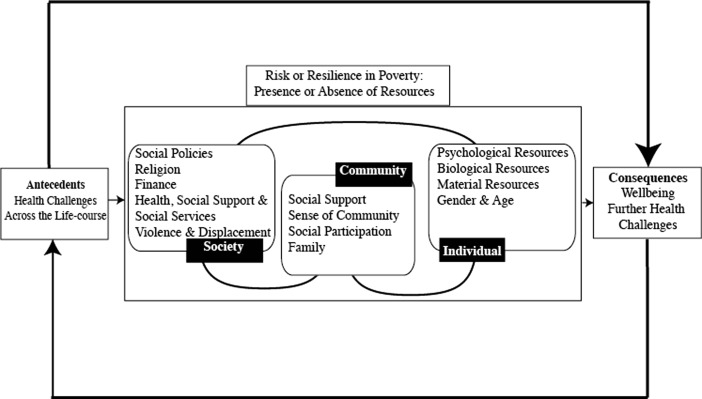
Ecological model of resilience in the context of poverty amongst older Colombians

The results demonstrate that, despite poverty, older Colombians in our sample are generally resilient. We are able to identify factors that contribute to resilience and we are able to place these factors within an ecological framework that functions at individual, community and societal levels. At the individual level, biological and material resources, alongside psychological characteristics, gender and age, contribute to resilience. At the community level, social support, participation and cohesion, and familial relationships are important. Finally at the societal level, social and welfare provision, finance, social policy and religion all play a part. However, the most significant aspect of the ecological model is the interplay between these levels. Whilst the resilience factors we identify map broadly onto our ecological framework (Fig. 1), they require modification. Figure 2 represents more effectively resilience in the context of older, poor Colombians, specifically.

In this modified framework, we demonstrate that individual characteristics are important for understanding resilience in this sample. Resilient participants are more likely to have material and biological resources to draw upon, such as physical health and some (relative) financial security. They are more likely to have a sense of mastery, personal control and optimism. However, these individual characteristics alone are rarely sufficient. Material resources are often contingent on the allowance provided by social policies such as the Project, or housing security dependent on the goodwill of others within the community. Psychological characteristics such as personal control can be undermined by wider social circumstances such as displacement. Community and societal factors alone are not sufficient to promote resilience. Without some individual contribution social policy is unable to make an individual resilient. It is the interplay of community and social levels that work most effectively. Without social policies such as the Project, social cohesion and social participation is less easily attained. Conversely, family roles contribute to wider societal structures, contributing to resilience. Thus, it is the collective combination of the individual, community and social levels that promotes resilience.

Additional factors contribute to resilience at all levels, such as age and gender, culture and religion. A person’s age and gender influences their individual behaviour and material and biological characteristics. However, the age and gender of an individual influences their place within a community and access to social support. At a societal level, social policy developments are influenced by societal attitudes towards age and gender. Both culture and religion affect an individual’s response to stressors. They may influence community responses, such as attendance at a local church. Finally, social policies are developed within a cultural framework that includes religious values. This data supports the idea that Latinos are, even in situations of poverty, resilient (Ong and Bergeman 2004; Gallo et al. 2009). The data also suggests that men and women respond differently to becoming older, and that this in turn impacts on resilience, with women seeing increased opportunities and men wishing they were younger. Thus, increased age in women might promote resilience but it might not do so in men. Religion is an important factor for both resilient and non-resilient participants. However, for those who are not resilient religion might be seen as a hindrance as it fostered passivity that might not be helpful.

This study has important implications for policy and practice. First, many older people in Colombia live in poverty, despite efforts to lift people out of poverty. Second, the study demonstrates the ways in which social policy interacts with the community, and by recognising this association, local and central government can develop policies to promote resilience. Third, it highlights the need for individual characteristics and resources to be accounted for when planning policies and interventions both at the societal and community levels. Fourth, it demonstrates how public policy in Colombia should prioritise older people’s access to public housing alongside the lack of work opportunities or pension provision amongst these people.

### How may Resilience Be Promoted in Older People Who Are not yet Resilient?

Older people living in Colombia are often poor. Six key features differentiated the non-resilient from the resilient: food insecurity; housing insecurity; poor health; lack of social participation; lack of social support; and lack of personal control. It is important at a societal level, therefore, to introduce policies to increase the standard of living for the older poor. Housing security impacts on resilience. Thus, the provision of housing security would be beneficial. Nevertheless, despite living in poverty the majority of participants are resilient. The Project itself is a policy that promotes resilience. Extending such projects promotes resilience. The Project also contributes to resilience at the community level. It not only provides financial assistance but also facilitates social support, cohesion and support. The personnel of the Project are important, as are the types of activities and support they provide. Further, the members of the Project provide mutual support. Thus, extending the reach of the Project would be beneficial at the community level. There is evidence that family support and solidarity is an important feature of resilience in Colombians. Policies that are designed to foster familial solidarity are valuable. The Project contributes at the individual level, promoting mastery, personal control and optimism. Policies could be introduced to enhance both material resources but also biological resources in terms of increasing health service availability. This research highlights the importance of considering policies and interventions to promote resilience within the ecological framework rather than at an individual level alone. Thus, none of the features displayed by the non-resilient participants appear to be unamenable to change through targeted intervention. Finally, the study provides some evidence for the argument that people should be encouraged to utilise available resources; that is, it is not only the availability but also the adoption of resources which is important.

### Limitations

There are some limitations to this study. First, the interviews were not aimed at collecting data on resilience per se, but on psychological wellbeing, so questions were not focused specifically on resilience. It is more striking, therefore, that resilience was the most salient theme in the data. Second, there are challenges in translating the data from Spanish to English; the direct translation of some phrases and idioms is not possible. However, as two of the researchers are native to Colombia, we consider the cultural features of the participants’ discourse in the analysis. Third, although the participants are living in poverty, they are connected with the Project, either actively or on a waiting list. This study, therefore, does not address resilience in those living in rural communities nor those in the most serious situations of deprivation and exclusion. Fourth, as we have mentioned previously, the fact that many of the participants were receiving an allowance or involved in the Project might contribute to resilience and thus act as confounding variable. Nevertheless, the evidence suggests that this was a necessary variable. The study was able to highlight a variety of factors and resources at different levels that contributed to resilience and were independent of the Project. An advantage of recruiting through the Project was that the criteria of poverty was met.

## Conclusion

The present research focuses on the little studied area of resilience in poverty, and on older Colombians in particular. Only Cárdenas and López (2010) have considered resilience in older Colombians and this study extends their work by placing resilience within an ecological framework, with a larger sample. Further, it addresses the ways in which older Colombians achieve resilience. Our findings make an important contribution to understanding resilience and in pointing to ways in which resilience can be enhanced in situations of poverty. The study has relevance to other parts of the developing world where poverty is common. In addition, the focus on Latinos has implications for Latinos living in other parts of the world including the USA. The study demonstrates how an ecological framework is useful in understanding how resilience can be achieved and the ways in which all three levels in the system (individual, community and societal) are interdependent. Finally the results show how public policies can be introduced to enhance the lives of older people living in poverty.
